# The Prediction of Early Neurological Outcomes in Out-of-Hospital Cardiac Arrest Patients: A Multicenter Prospective Cohort Study by the KORHN Registry

**DOI:** 10.3390/jcm14186466

**Published:** 2025-09-13

**Authors:** Wook Jin Choi, Jae Hoon Lee

**Affiliations:** 1 Department of Emergency Medicine, University of Ulsan College of Medicine, Ulsan 44033, Republic of Korea; koreanermd@gmail.com; 2 Department of Emergency Medicine, Dong-A University College of Medicine, Busan 49201, Republic of Korea

**Keywords:** out-of-hospital cardiac arrest, hypothermia, induced, patient outcome assessment, risk factors

## Abstract

**Background/Objectives**: Early neuroprognostication after cardiac arrest is essential for guiding treatment strategies and providing accurate prognostic information to families. While several early risk scores have been proposed, few have incorporated a wide range of variables in large cohorts. This study aimed to develop and validate a novel prognostic model, the KORHN risk score, and to compare its performance with established tools including MIRACLE, TTM, CAHP, C-GRApH, and OHCA scores; **Methods**: We conducted a prospective multicenter observational study using data from the KORean Hypothermia Network registry. Risk variables identified in previous studies, along with extensive data from 1371 patients in the KORHN registry, were analyzed. The primary endpoint was poor neurological outcome at 6 months; **Results**: Key predictors included low-flow time, diastolic shock index, cardiac etiology, bilateral absence of pupil reflex, shockable initial rhythm, Glasgow Coma Scale motor response, epinephrine use, and age. Compared with established risk scores, the KORHN score demonstrated superior performance (AUC 0.925 vs. 0.827–0.902 with all variables, and AUC 0.914 vs. 0.85–0.903 with the top five variables with identical cut-off). External validation in a non-KORHN cohort (AUC 0.890) confirmed its robustness; **Conclusions**: The KORHN score provides a simple, accurate tool for early neuroprognostication, supporting clinical decision-making and family communication.

## 1. Introduction

Early prognostication in patients with cardiac arrest is crucial for risk assessment. Initial risk analysis is necessary not only to guide decisions regarding the use of high-cost intensive treatments at admission, but also to enable more precise discussions with patients’ families about prognosis. As risk stratification is already employed to guide management in conditions such as stroke and myocardial infarction [1,2], there is an emerging need for risk-based management strategies in patients with cardiac arrest as well. Risk stratification can be initially assessed through risk scores, biomarkers, brain MRI, and electroencephalography (EEG). Several representative risk scores, such as MIRACLE2, TTM, CAHP, C-GRApH, and OHCA [3,4,5,6,7], have been developed. Prognostic prediction using brain MRI within the first 6 h and frontal EEG on the day of admission have also been reported as meaningful [8,9]. There is a pressing need for validated early risk-based management strategies that integrate these approaches.

To date, large randomized controlled trials have found no significant advantages of targeted temperature management (TTM) at 33 °C compared to 36 °C [10]. However, multiple studies have demonstrated that TTM at 33 °C can provide survival benefits in patients with moderate ischemic brain injury [11,12,13,14]. Patients with mild encephalopathy who exhibited normal continuous electroencephalogram patterns at 12 h post-cardiac arrest and those with severe encephalopathy characterized by suppressed background activity did not benefit from TTM at 33 °C. However, the therapeutic effects of TTM at 33 °C were observed in patients with moderate encephalopathy resulting from cardiac arrest [12]. Therefore, determining the target temperature of TTM based on initial risk assessment may be a useful approach.

Since TTM is not entirely physiologic—it may reduce heart rate (HR) and cardiac output [15] and increase the risk of infection [16]—TTM should be selectively applied only in patients with moderate brain injury, excluding those with mild brain injury or confirmed brain death.

For these reasons, we aimed to develop a tool for early risk assessment in cardiac arrest patients. Our goal was to predict early neuroprognostication using basic clinical data that are readily available in most hospitals, where modalities such as brain MRI or EEG are less accessible in the early phase. We sought to develop a newly established risk score that can be used for clinical risk stratification and to compare its performance with that of existing risk scores.

## 2. Materials and Methods

### 2.1. Study Design and Setting

The KORHN registry for cardiac arrest is a prospective, multicenter, observational cohort study conducted using a clinical research consortium for TTM in Korea. Data were collected from patients with out-of-hospital cardiac arrest who underwent TTM in 22 teaching hospitals. TTM was performed primarily at 33 °C, with the optional adjustment to 34–36 °C for hemodynamic instability. This temperature was maintained for 36 h followed by gradual rewarming at a rate of 0.1 °C/h. Each hospital obtained study approval and registered it with the International Clinical Trials Registry Platform (ClinicalTrials.gov identifier: NCT02827422). Dong-A University Hospital’s Institutional Review Board approved this study under entry code DAUHIRB-16-079 (Approval Date: 27 April 2016). The study at our institution was initiated after obtaining IRB approval, and the participating hospitals began collecting data following the approval of their respective IRBs. The investigations were conducted in accordance with the principles outlined in the Declaration of Helsinki (1975, revised in 2013). According to point 23 of this declaration. Informed consent was obtained from the legal surrogates of all patients in accordance with national requirements and the Declaration of Helsinki. The data were systematically reviewed on a regular basis by a team comprising clinical research associates, investigators, and clinical research coordinators at each participating site. Prior to the study, all centers were provided with the same aggressive TTM protocol but maintained autonomy over vasoactive drugs, fluid management, and rewarming protocols. The novel KORHN score and key variables were externally validated using a dataset from a separate institution not included in the KORHN registry.

### 2.2. Study Population

Among 10,258 patients who experienced cardiac arrest, 1372 comatose patients who underwent targeted temperature management (TTM) were included in the study. Inclusion criteria were as follows: age greater than 18 years, underwent TTM after out-of-hospital cardiac arrest, and unconsciousness defined as a Glasgow Coma Scale (GCS) score below 8 after return of spontaneous circulation (ROSC). Exclusion criteria were as follows: rearrest during the initial 24 h following admission, acute ischemic or hemorrhagic stroke, treatment refusal, do-not-resuscitate order, pre-arrest neurological disability (cerebral performance category [CPC] 3–4), clinical conditions limiting survival to six months or less, and a body temperature of 30 °C or below on admission. A total of 1372 cardiac arrest patients were divided into two groups for analysis: 428 patients with a good prognosis and 943 patients with a poor prognosis. The results of the analysis were validated through external validation. The external validation dataset included 314 patients who survived a cardiac arrest at Ulsan University Hospital in Korea.

### 2.3. Variables and Endpoints

The KORHN registry collected demographic (age, sex, BMI, pre-hospital ECG pattern, previous history), resuscitation (witness arrest, bystander CPR, no-flow time, low-flow time, time from ROSC to TTM start, epinephrine dose), and post-resuscitation data (blood pressure, HR, pupil reflex, pH, PaCO_2_, lactate, creatinine, glucose), as well as risk variables upon emergency department admission from previous studies. By comparing previously established risk scores, the optimal predictors were determined. In addition to previous established variables, cardiac arrest due to a cardiac etiology, HR, diastolic arterial pressure (DAP), pre-CPC and MRC scores, full outline of unresponsiveness (FOUR), and cardiovascular sequential organ failure assessment (SOFA) scores were evaluated for early neurological prognostication. The data were evaluated to develop a new score (KORHN score), compared to the MIRACLE2, TTM, CAHP, C-GRApH, and OHCA scores [3,4,5,6,7]. The analysis was performed on complete cases, excluding any missing data. The main endpoint was unfavorable neurological outcome at 6 months after admission, defined as CPC scores of 3–5, determined from medical records and follow-up calls with transfer hospitals.

### 2.4. Statistical Analysis

Both univariate and multivariate analyses were performed to identify significant predictors of poor neurological outcomes. Categorical variables were compared using Fisher’s exact test, and continuous variables were compared using the Mann–Whitney U test. All statistically significant variables were standardized, followed by a multivariable binary logistic regression analysis to identify significant covariates and calculate adjusted odds ratios for early prognosis. The best combination of pivotal predictors was evaluated, and changes in risk associated with these predictors were examined using a predictive function. This function forecasted poor neurological outcomes, and its performance was visualized through cubic spline plots. We compared the KORHN score with other risk scores by identifying the top five significant variables from previously proposed risk scoring tools. All continuous variables that were used in the analysis of this five-value set were converted into categorical variables. The cut-off values for these variables were determined using the Youden index to balance the ORs. Firstly, the original risk variables for neuroprognostication in previous studies were validated again using this dataset. The risk scores’ capacity to distinguish poor neurological outcomes was assessed using the area under the receiver operating characteristic curve (AUC) and was calculated to evaluate sensitivity, specificity, and positive and negative predictive values.

## 3. Results

### 3.1. Patient Characteristics and Poor Neurological Outcomes

Out of the 1372 patients included in the study, 429 (31.2%) had good neurological outcomes. Table 1 shows factors associated with poor outcomes, including old age, male sex, unwitnessed arrest, bystander cardiopulmonary resuscitation, prolonged low-flow time, pre-hospital non-shockable rhythm, specific electrocardiogram patterns, cardiac and non-cardiac cause of cardiac arrest, unreactive pupils, low GCS motor response, low FOUR score, SOFA score, increased epinephrine use and abnormal levels of pH, PaCO_2_, lactate, creatinine, and glucose levels.

### 3.2. Key Variables and the KORHN Score

**Figure 1:**
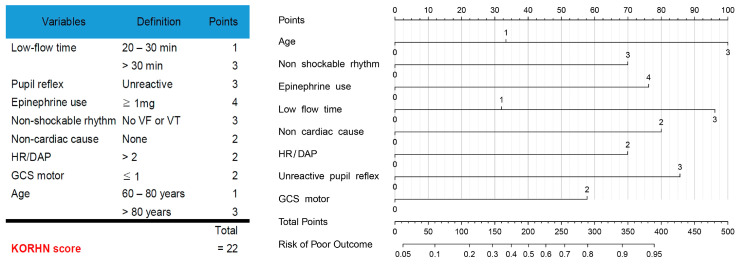
The KORHN risk score and nomogram. HR/DAP, heart rate/diastolic pressure; KORHN, Korean Hypothermia Network; VF, ventricular fibrillation; VT, ventricular tachycardia. GCS, Glasgow Coma Scale.

**Figure 2:**
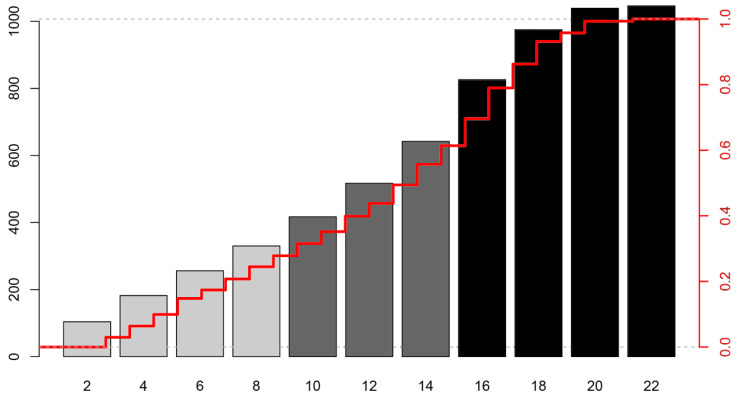
Cumulative distribution on risks of poor neurologic outcomes according to the KORHN risk score. KORHN, Korean Hypothermia Network.

All meaningful variables were standardized and compared using adjusted odds ratios; Table 2 ranks these significant variables. The KORHN score was developed by extracting the eight most important variables. Low-flow time > 21 min, HR/DAP ratio > 1.7, non-cardiac cause, unreactive pupil reflex, non-shockable rhythm, GCS motor score ≤ 1, administration of epinephrine at least once, and age > 66 years were associated with adjusted odds ratios of 4.287, 3.523, 3.438, 3.393, 3.087, 2.614, 2.526, and 2.419, respectively, with all *p*-values less than 0.001 (Table 2). For these key variables, inflection points or sharp changes were identified using cubic spline plots to determine cut-off values, and multiple ROC curve analyses were performed to detect the optimal points. The KORHN score incorporates these decisively determined key variables. The score consists of 22 points in total, with each variable assigned between 1 and 4 points (Figure 1). For interpretation, each variable is converted to a standardized score out of 100, and the total score is used in a nomogram to predict the risk probability of poor neurological outcome (Figure 1). The KORHN score also demonstrates a sequential increase in risk as the score rises, as illustrated by the cumulative distribution (Figure 2). A cardiac arrest patient presented with the following characteristics: age 65 years, ventricular fibrillation rhythm, epinephrine administered twice during CPR, low-flow time of 22 min, cardiac arrest following chest pain, HR of 95 beats per minute, diastolic arterial pressure of 58 mmHg within 1 h post-ROSC, absent pupil reflex, and GCS motor score of 1. The corresponding KORHN scores (converted scores) are as follows: age 1 point (33.3), non-shockable rhythm 0 points (0), epinephrine use 4 points (76.3), low-flow time 1 point (32) [3 points would correspond to 96.2], cardiac cause 0 points (0), HR/DAP 0 points (0), pupil reflex 3 points (86.7), and GCS motor 2 points (57.7). The total converted score sums to 286 points. Drawing a vertical line from 286 on the nomogram corresponds to a risk of poor outcome of 0.79, indicating a 79% probability of a CPC score of 3 or higher.

### 3.3. Comparison of the KORHN Score and Other Risk Scores

**Figure 3:**
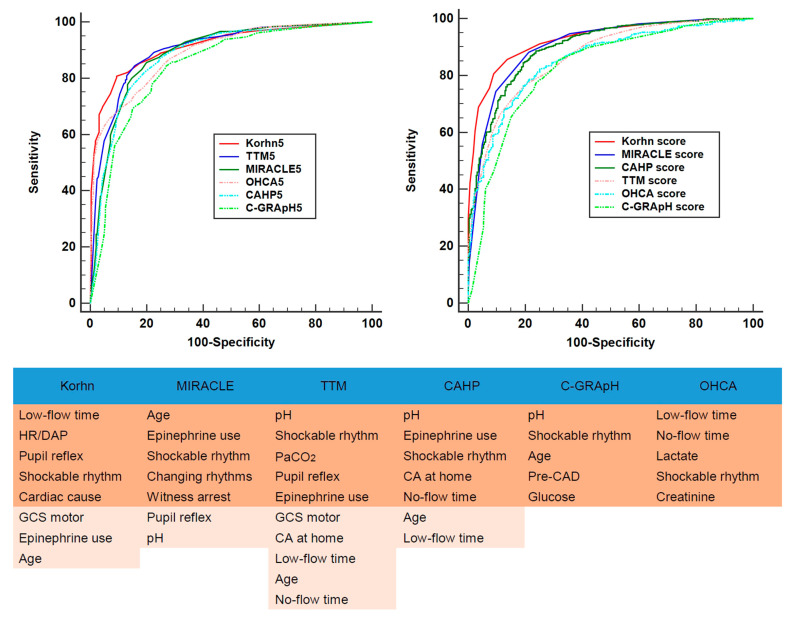
Components and performances of risk scores. KORHN, Korean Hypothermia Network; HR/DAP, heart rate/diastolic pressure; CA, cardiac arrest; CAD, coronary artery disease.

While the KORHN score consists of eight variables, previous risk scores have used between five and ten variables (Figure 3). In earlier studies, the CAHP score demonstrated the highest predictive power (sensitivity 87.1%, specificity 67.1%, AUC 0.93), but when compared to other risk scores using the KORHN registry, the MIRACLE score exhibited superior performance (sensitivity 88.82%, specificity 77.95%, AUC 0.902) (Table 3). The C-GRApH score showed the lowest performance (sensitivity 76.92%, specificity 76.34%, AUC 0.827). KORHN scores greater than 9 outperformed the other risk scores in predicting poor neurological outcomes (AUC 0.925 vs. 0.827–0.902). When the top five variables had identical cut-off values, the KORHN score exhibited the highest performance compared to other risk scores (AUC 0.914 vs. 0.85–0.903) (Figure 3 and Table 3). In external validation with a small population, the KORHN score yielded an AUC of 0.890 (95% confidence interval, 0.85–0.92).

## 4. Discussion

### 4.1. The Overall Results

Among the numerous variables examined, low-flow time, diastolic shock index, non-cardiac-origin arrest, absent pupillary light reflex, non-shockable rhythm, motor response on GCS, epinephrine administration, and age demonstrated strong associations with poor neurological outcomes. The KORHN score demonstrated better performance compared to other risk scores. When compared not only to the main variables but also to the top five variables used in all risk scores, the KORHN risk score, which incorporates these promising predictors, demonstrated superior performance compared to the existing risk scores.

### 4.2. Promising Variables of the KORHN Score

The high performance of the KORHN score may be attributable to several factors. Firstly, significant variables were carefully selected from among a large pool, considering their interactions. This comprehensive search and consideration of inter-variable effects likely allowed for the identification of more relevant and important predictors. Secondly, scoring of the significant variables was weighted using cubic spline plots to enhance statistical discrimination. Employing cubic spline plots for setting multiple cut-off values and assigning points according to risk distribution proved highly effective. Thirdly, the KORHN score incorporated previously unused factors such as cardiac arrest due to cardiac cause, hypotension, and HR as key variables—items not included in other risk scores. The inclusion of these factors, identified through literature review and data analysis, likely improved prognostic performance.

Low-flow time is a well-established prognostic factor for cardiac arrest patients. Previous studies have shown that the median low-flow time was significantly prolonged in patients who experienced poor neurological outcomes (15 vs. 7.5 min, *p* < 0.0001; 25.7 min, *p* < 0.001; 27 vs. 19 min, *p* < 0.0001, respectively) [4,5,6]. Low-flow times between 15 and 27 min were correlated with KORHN scores of 0 or 1. In contrast, the median low-flow time in our cohort exceeded 30 min, aligning with a higher KORHN score of 3. This suggests that our study included a higher proportion of severely ill patients compared to previous studies. The reason no-flow time is not a strong prognostic factor may be due to uncertainties in determining the actual duration of cardiac arrest.

DAP or the diastolic shock index may better reflect vasodilation in complex shock states than systolic arterial pressure or mean arterial pressure [17]. Furthermore, in cardiac arrest patients, HR and DAP have been shown to be powerful predictors of poor neurological outcomes. Previous studies have reported that a higher HR after ROSC [18,19] and lower DAP [20,21] were both associated with unfavorable neurological outcomes. Although these were not included in previous early risk scores, they are now recognized as important predictors.

Cardiac arrest resulting from a cardiac cause and the presence of a shockable rhythm are favorable prognostic indicators because they offer the potential for reversible treatment. Key interventions such as cardioversion, coronary reperfusion, or the administration of diuretics can address underlying conditions such as ventricular tachyarrhythmia, acute coronary syndrome, or heart failure. In contrast, cardiac arrest due to non-cardiac causes or non-shockable rhythms is much less amenable to reversal [22,23]. Systematic chart reviews revealed that only 26% of cases were truly cardiogenic, significantly lower than previous estimates, and that a substantial proportion were due to respiratory failure (15%) or toxicological causes (9%). This highlights the prevalence of misclassification as cardiac events in the early phase [24]. One study found 89% concordance between initial suspected and final confirmed causes, indicating the difficulty in accurately categorizing cardiac etiology immediately on admission [25]. The potential inaccuracy in diagnosing cardiac arrest due to a cardiac cause may affect the accuracy of scoring systems; however, it remains an important prognostic predictor.

Neurological examination proved to be a strong prognostic indicator. In our study, pupil reflex, the GCS, and FOUR scores were evaluated, with pupil reflex demonstrating the most prognostic value. The odds ratio for the pupil reflex in our study was 3.39, compared to odds ratios of 2.49 (MIRACLE2) and 2.46 (TTM score) in previous research [3,4]. This difference may be due to our larger sample size (1371 vs. 373 and 933), the inclusion of a greater number of covariates, and a higher proportion of patients with poor neurological outcomes (68.7% vs. 59.8% and 52%). The assessment of pupil reflex is also subject to potential inaccuracies; its accuracy may be affected by sedative use or failure to detect subtle changes in pupil reactivity. Nevertheless, because it is an important prognostic indicator, efforts should be made to ensure accurate measurement. Establishing standardized protocols would be beneficial by considering methods using pupillometry, approaches that involve repeated measurements, and strategies to withhold pupil reflex assessments in cases of miosis—taking into account the possibility of opioid-induced miosis.

### 4.3. Comparison Between the KORHN Score and Other Risk Scores

The performance of risk scores may vary depending on the patient population studied. Consistent with our findings, the C-GRApH score has demonstrated slightly lower performance compared to other risk scores in cardiac arrest patients [26,27]. In a meta-analysis including seven risk scores, the CAHP score was identified as the most accurate discriminator for early neuroprognostication [28]. However, OHCA, CAHP, TTM, and MIRACLE scores have exhibited comparable performance (AUC 0.82 to 0.88), with varying rankings in terms of superiority [4,27,29,30,31].

To our knowledge, previous studies have not simultaneously compared promising risk scores, including the OHCA, CAHP, and MIRACLE scores, for early prognostication, nor has any study included a population larger than ours. The current study included a wide range of variables, enabling comparison and evaluation of prior risk scores. Furthermore, we standardized the number of variables considered (top five) for direct comparison, potentially increasing the precision of the assessment. Reported AUC values in earlier studies ranged from 0.82 to 0.93, but within our larger dataset, the KORHN score demonstrated the highest performance. Although this may be partly attributable to the score’s derivation from our own dataset, it is clear that the KORHN score is as effective and predictive as the MIRACLE, TTM, CAHP, and OHCA scores for early outcome prediction.

### 4.4. Clinical Implementation

An automated system may be required for clinical application. A short form to record low-flow time and checkboxes for pupil reflex, motor response, epinephrine use, defibrillation performed, and cardiac cause should be created on clinical charts and nurse input forms. Patient age, HR, and DAP within 1 h of ROSC can be extracted automatically from electronic medical records to calculate and display the KORHN risk score. Using this score, an algorithm could be developed to initiate TTM for patients with a score of 9 or higher. Decisions regarding the use of treatments such as TTM or extracorporeal membrane oxygenation in patients with very high scores will require further discussion. Additional early EEG [9] or brain MRI [8] may assist in these clinical decisions.

## 5. Limitations

This study has several limitations. Firstly, although the internal validation cohort included the largest sample, the external validation involved a relatively small population, necessitating further verification of the KORHN risk score with components from other risk scores. Secondly, medication and ventilator settings varied between institutions according to clinical needs, which may have influenced neurological outcomes. Thirdly, we did not consider post-hospital interventions or EEG, as neuroprognostication should be conducted early and applicable in general medical settings. Particularly, early EEG analysis using the mean suppression ratio can be applied to critical prognostic assessments in cardiac arrest patients [9], and utilizing this method could further enhance the predictive power of the KORHN risk score. Fourthly, measurement of pupil reflex may have been affected by sedative use or by missing subtle changes. Fifthly, despite being a retrospective analysis of a prospectively collected nationwide registry, there may have been inaccuracies in classifying the etiology of cardiac arrest.

## 6. Conclusions

In conclusion, the KORHN score is an effective risk scoring system, comparable to existing risk scores. It is a simple and reliable tool incorporating low-flow time, pupillary light reflex, epinephrine administration, shockable cardiac rhythm, cardiac-origin cardiac arrest, HR/DAP, motor response on GCS, and age to determine early neuroprognostication in out-of-hospital cardiac arrest patients upon admission. Early risk stratification using the KORHN score may assist in therapeutic decision-making and family counseling for cardiac arrest patients. However, due to the small sample size for external validation, generalization is challenging, and further validation in diverse populations, including non-Asian cohorts, is necessary to confirm broader applicability.

## Figures and Tables

**Table 1:** Baseline characteristics at the early stage.

Variable	All Patients (*n* = 1371)	Good Outcome (*n* = 428)	Poor Outcome (*n* = 943)	*p* Value ^1^
Age, years	62 (51–74)	58 (48–66)	65 (53–77)	<0.001
Male, *n* (%)	975 (71.1)	333 (77.8)	642 (68.1)	<0.001
BMI, kg/m^2^	23.3 (20.9–25.7)	23.3 (21.3–25.6)	23.4 (20.8–25.7)	0.823
Witness arrest, *n* (%)	949 (70)	361 (84.5)	588 (63.3)	<0.001
Bystander CPR, *n* (%)	843 (62.4)	292 (69.2)	551 (59.3)	0.001
Time from arrest to CPR start, minutes	1 (0–7)	1 (0–5)	1 (0–8)	0.005
Time from CPR start to ROSC, minutes	15 (9–22.75)	15 (9–22.8)	31 (20–42)	<0.001
Time from ROSC to TTM start, hours	3.4 (2.2–4.9)	3.6 (2.5–5)	3.3 (2–4.8)	0.002
Pre-hospital ECG rhythm				<0.001
Asystole, *n* (%)	445 (37)	23 (6.1)	422 (51)	
PEA, *n* (%)	269 (22.3)	54 (14.3)	215 (26)	
Pulseless VT, *n* (%)	15 (1.2)	11 (2.9)	4 (0.5)	
VF, *n* (%)	448 (37.2)	272 (72.1)	176 (21.3)	
ROSC, *n* (%)	27 (2.2)	17 (4.5)	10 (1.2)	
Baseline ECG findings				<0.001
ST segment elevation, *n* (%)	169 (12.5%)	75 (17.7%)	94 (10.2%)	
ST segment depression, *n* (%)	250 (18.5%)	75 (17.7%)	175 (18.9%)	
Bundle branch block, *n* (%)	263 (19.5%)	59 (13.9%)	204 (22.1%)	
Unspecific finding, *n* (%)	666 (49.4%)	215 (50.7%)	451 (48.8%)	
Past medical history				
Cardiovascular disease ^2^, *n* (%)	285 (20.8)	99 (34.7)	186 (19.7)	0.152
Neurologic disease ^3^, *n* (%)	138 (10.1)	27 (6.3)	111 (11.8)	0.002
Respiratory disease, *n* (%)	106 (7.7)	13 (3)	93 (9.9)	<0.001
Cancer, *n* (%)	80 (5.8)	23 (5.4)	57 (6)	0.465
Psychiatric disorder, *n* (%)	51 (3.7)	5 (1.2)	46 (4.9)	<0.001
Cardiac-origin cardiac arrest, *n* (%)	850 (62)	372 (86.9)	478 (50.7)	<0.001
Cardiac arrest etiology, *n* (%)				<0.001
Medical causes, *n* (%)	851 (62.1)	479 (50.8)	372 (86.9)	
Traumatic causes, *n* (%)	28 (2)	2 (0.5)	26 (2.8)	
Submersion injury, *n* (%)	19 (1.4)	4 (0.9)	2 (0.2)	
Electrical injury, *n* (%)	6 (0.4)	3 (0.5)	3 (0.4)	
Toxicological cause, *n* (%)	16 (1.2)	5 (1.2)	11 (1.2)	
Hypoxic injury, *n* (%)	78 (5.7)	6 (1.4)	72 (7.6)	
Strangulation, *n* (%)	160 (11.7)	12 (2.8)	148 (15.7)	
Miscellaneous causes, *n* (%)	213 (15.5)	23 (5.4)	190 (20.1)	
Pre-cardiac arrest CPC score	1 (1–1)	1 (1–1)	1 (1–1)	<0.001
Pre-cardiac arrest MRS	0 (0–1)	0 (0–0)	0 (0–1)	<0.001
Pupillary light reflex, *n* (%)	643 (47.3)	346 (81)	297 (31.8)	<0.001
Motor response on GCS, score	1 (1–1)	1 (1–3)	1 (1–1)	<0.001
Four-scale score ^4^	0 (0–3)	4 (0–7)	0 (0–1)	<0.001
Cardiovascular SOFA ^5^	4 (2–4)	3 (0–4)	4 (3–4)	<0.001
Diastolic shock index	1.41 (1.1–1.9)	1.25 (1–1.6)	1.5 (1.1–2.1)	<0.001
Epinephrine administration dose, mg	2 (0–4)	0 (0–1)	2 (1–4)	<0.001
pH	7.08 (6.92–7.23)	7.22 (7.09–7.3)	7.02 (6.89–7.17)	<0.001
PaCO_2_, mmHg	108.9 (72.7–196.8)	108.8 (73.7–192.7)	109.4 (72–199.8)	<0.001
Lactate, mg/dL	9.7 (6.1–12.9)	7.1 (4.3–10.9)	10.6 (7.5–13.6)	<0.001
Creatinine, mg/dL	1.3 (1.1–1.8)	1.2 (1–1.4)	1.4 (1.1–2.2)	<0.001
Glucose, mg/dL	255 (190–330)	238 (180–297)	266 (197–347)	<0.001

Values are expressed as number (%) or median (interquartile range). BMI denotes body mass index; CPR, cardiopulmonary resuscitation; ROSC, restoration of spontaneous circulation; TTM, targeted temperature management; PEA, pulseless electric activity; VT, ventricular tachycardia; VF, ventricular fibrillation; ECG, electrocardiography; CPC, cerebral performance category; MRS, modified Rankin scale; GCS, Glasgow coma scale. ^1^ The exact Fisher test and the Mann–Whitney nonparametric test were used for the *p* value. ^2^ The spectrum of cardiovascular disease covered heart failure, coronary artery disease, and cardiac arrest. ^3^ Neurological disease included diseases such as transient ischemic accident, stroke, and other neurological diseases. ^4^ The score, ranging between 0 and 4, evaluated eye response, motor response, brainstem reflexes, and respiration. ^5^ Cardiovascular SOFA scores ranged from 0 to 4, defined as follows: 0 indicates no hypotension; score 1 corresponds to a mean arterial pressure (MAP) below 70 mmHg; score 2 indicates the use of dopamine at ≤5 µg/kg/min; score 3 represents epinephrine norepinephrine at ≤0.1 µg/kg/min or dopamine > 5 µg/kg/min or dobutamine at any dose; and score 4 corresponds to epinephrine or norepinephrine > 0.1 µg/kg/min or dopamine > 15 µg/kg/min.

**Table 2:** Significant risk factors determined by multivariable analysis.

Variable	Β-Coefficient	Adjusted OR	*p* Value	95% CI
Low-flow time > 21 min	1.455	4.287	<0.001	2.62–7.013
HR/DAP > 1.7	1.259	3.523	<0.001	2.037–6.095
Non-cardiac-origin arrest	1.235	3.438	<0.001	2.097–5.49
Absent pupillary light reflex	1.222	3.393	<0.001	1.82–6.496
Non-shockable rhythm	1.127	3.087	<0.001	1.842–5.175
Motor response on GCS ≤ 1	0.961	2.614	<0.001	1.579–4.328
Epinephrine administration	0.927	2.526	<0.001	1.539–4.144
Age > 66 years old	0.883	2.419	<0.001	1.518–3.854
Creatinine > 1.4 mg/dL	0.814	2.257	0.001	1.38–3.692
Unwitnessed arrest	0.802	2.231	0.007	1.24–4.014
Lactate > 8.2 mg/dL	0.671	1.957	0.004	1.237–3.095
ST elevation	0.652	1.919	0.037	1.039–3.545
PaCO_2_ > 48 mmHg	0.628	1.875	0.008	1.176–2.989

All included variables were standardized if statistically significant. Cut-off values for continuous variables were determined using Youden’s index. HR/DAP, heart rate/diastolic pressure; GCS, Glasgow Coma Scale.

**Table 3:** Comparison of predictive performance among risk scores.

	N	Sensitivity	Specificity	PPV	NPV	AUC
Performance in previous data ^1^						
MIRACLE > 2	373	97.9	56.7	77.9	94.4	0.9
TTM > 10	933	92.6	61.4	78.7	84.3	0.842
CAHP > 150	819	87.1	67.1	81	76.4	0.93
OHCA > 2	210	96.9	34.7	71.1	87.2	0.82
Performance in the present data						
KORHN score > 9	1045	86.01	85.44	93.4	71.9	0.925
MIRACLE score > 3	1082	88.82	77.95	90.5	74.7	0.902
TTM score > 12	1054	93.6	51.32	82.6	76.5	0.863
CAHP score > 0.57	1072	88.22	76.3	90.2	72.3	0.894
OHCA score > 12.65	1141	77.09	80.34	89.8	60.9	0.853
C-GRApH score > 0.98	1148	76.92	76.34	87.9	59.7	0.827
Performance based on the top 5 variables using a fixed threshold						
KORHN5	1139	80.56	90.2	94.7	67.9	0.914
TTM5	1054	84.53	84.21	93	68.8	0.903
MIRACLE5	1082	86.32	79.19	90.7	71	0.89
OHCA5	1141	82.41	76.64	88.8	68.9	0.889
CAHP5	1072	86.65	75.65	89.8	69.6	0.881
C-GRApH5	1148	83.48	74.65	88	66.9	0.85

^1^ Multiple imputation methods were utilized in the MIRACLE and TTM studies for missing data, but there was no mention of missing data handling in the CAHP and OHCA studies. KORHN, Korean Hypothermia Network; PPV, positive predictive value; NPV, negative predictive value; AUC, area under the receiver operating characteristic curve.

## Data Availability

The data presented in this study are available on request from the corresponding author.

## Abbreviations

EEG: electroencephalography; TTM: targeted temperature management; HR: heart rate; GCS: Glasgow Coma Scale; ROSC: restoration of spontaneous circulation; CPC: cerebral performance category; DAP: diastolic arterial pressure; FOUR: full outline of unresponsiveness; SOFA: sequential organ failure assessment; AUC: the area under the receiver operating characteristic curve.
